# Applying the exposome concept in birth cohort research: a review of statistical approaches

**DOI:** 10.1007/s10654-020-00625-4

**Published:** 2020-03-27

**Authors:** Susana Santos, Léa Maitre, Charline Warembourg, Lydiane Agier, Lorenzo Richiardi, Xavier Basagaña, Martine Vrijheid

**Affiliations:** 1 The Generation R Study Group, Erasmus MC, University Medical Center Rotterdam, PO Box 2040, 3000 CA Rotterdam, The Netherlands; 2 Department of Pediatrics, Erasmus MC – Sophia Children’s Hospital, University Medical Center Rotterdam, Rotterdam, The Netherlands; 3 Institute for Global Health, ISGlobal, Barcelona, Spain; 4 Universitat Pompeu Fabra (UPF), Barcelona, Spain; 5 CIBER Epidemiología y Salud Pública (CIBERESP), Barcelona, Spain; 6 Team of Environmental Epidemiology, IAB, Institute for Advanced Biosciences, Inserm, CNRS, CHU-Grenoble-Alpes, University Grenoble-Alpes, Grenoble, France; 7 Cancer Epidemiology Unit, Department of Medical Sciences, University of Turin and CPO-Piemonte, Turin, Italy

**Keywords:** Exposome, Birth cohorts, Life-course epidemiology, Omics, Environmental epidemiology

## Abstract

The exposome represents the totality of life course environmental exposures (including lifestyle and other non-genetic factors), from the prenatal period onwards. This holistic concept of exposure provides a new framework to advance the understanding of complex and multifactorial diseases. Prospective pregnancy and birth cohort studies provide a unique opportunity for exposome research as they are able to capture, from prenatal life onwards, both the external (including lifestyle, chemical, social and wider community-level exposures) and the internal (including inflammation, metabolism, epigenetics, and gut microbiota) domains of the exposome. In this paper, we describe the steps required for applying an exposome approach, describe the main strengths and limitations of different statistical approaches and discuss their challenges, with the aim to provide guidance for methodological choices in the analysis of exposome data in birth cohort studies. An exposome approach implies selecting, pre-processing, describing and analyzing a large set of exposures. Several statistical methods are currently available to assess exposome-health associations, which differ in terms of research question that can be answered, of balance between sensitivity and false discovery proportion, and between computational complexity and simplicity (parsimony). Assessing the association between many exposures and health still raises many exposure assessment issues and statistical challenges. The exposome favors a holistic approach of environmental influences on health, which is likely to allow a more complete understanding of disease etiology.

## Introduction

Disease risk is largely determined by behavioral, environmental, and occupational risk factors [1]. Many of these risk factors are modifiable and therefore can be a potential target for prevention. However, current insights into the association between many of these risk factors and health are too limited for effective prevention. A substantial proportion of the modifiable risk of major chronic diseases remains to be discovered [1]. Also, not much is known about the complex interrelations between risk factors and how they relate to biological endogenous responses. The holistic exposome concept may provide a useful framework to broaden our understanding of the impact over the life course of a multitude of risk factors on human health, and ultimately contribute to explaining the unattributable burden of disease and developing effective preventive measures. Pregnancy and birth prospective cohorts, combining a wealth of data collected at the individual and population level by methods such as questionnaires, physical examinations, biological samples and geo-spatial modelling, represent a unique and resourceful opportunity to perform exposome studies of the fetal and early life onwards.

In this paper, we describe the steps required for applying an exposome approach in epidemiologic studies, with a specific focus on prospective birth cohort studies. We intend to provide researchers with relevant information to perform exposome analyses, understand the main strengths and limitations of different statistical approaches and list the challenges for future research on the exposome.

## The exposome

The concept of the exposome was first proposed in 2005 by the cancer researcher Christopher Wild to encompass ‘life-course environmental exposures (including lifestyle factors), from the prenatal period onwards' [2]. It was developed to highlight the need for more accurate, reliable and comprehensive environmental exposure data to complement the impressive advances made in measuring the human genome. The exposome has been proposed to comprise three overlapping and complementary domains: (1) a general external domain, including factors such as climate, urban/rural environment and societal factors, which are mainly assessed at the community level by geographical mapping methods; (2) a specific external domain, including environmental pollutants, tobacco and diet, which are assessed at the individual level by questionnaires or biomonitoring, and (3) an internal domain, including internal body processes such as inflammation, metabolism, and endogenous circulating hormones, which are often assessed by high-throughput molecular omics methodologies [3]. The internal exposome is expected to, at least partly, reflect the external exposome. The exposome is complex and challenging in many respects. Measuring it implies the accurate and reliable assessment of many time-varying exposures over the life course. The technical challenges of measuring the external and internal domains of the exposome are beyond the scope of this review and have been discussed elsewhere [4, 5]. Continuous exposome assessment is difficult and thus the life course exposome is usually obtained from exposure assessments at specific time points. The choice of the time points is of utmost importance since the health effects of a given exposure may vary during the various developmental periods or may be altered when it co-occurs with other exposures through synergistic/interactive effects [6]. Since the developing fetus is particularly vulnerable to the effects of environmental exposures and since adverse exposures in utero during critical windows may have a lifetime health impact, the pregnancy period is an important starting point to develop a lifetime exposome [6]. Other possible key time points where measures of the exposome could be made include the infancy, childhood, adolescence and adulthood life periods [3]. Whilst it is clear that full measurement of the exposome at even a single time point is currently impossible, even partial exposome coverage, where neither the totality of exposures nor the dynamic coverage are assessed, is valuable [3]. Important progresses in uncovering the various aspects of the exposome are currently being made by different research projects worldwide, including the LifeCycle (www.lifecycle-project.eu), HELIX (www.projecthelix.eu), EXPOsOMICS (www.exposomicsproject.eu), HEALS (www.heals-eu.eu), HERCULES (https://emoryhercules.com) and CHEAR (https://chearprogram.org) projects [7–11]. Further, an R package called rexposome, which allows exposome data loading, exploration, and analysis has been developed [12]. In the next sections of this paper, we describe the main steps undertaken in exposome research.

## Selecting and pre-processing exposures

The selection of exposures to consider in the study depends on the research question and issues of feasibility and data accessibility. Previous studies have included a wide range of external environmental exposures from various families, or have been specifically focused on certain components of the exposome, such as the urban or the chemical exposome, assessed at various developmental periods [13–19]. Socio- and public health exposome conceptual frameworks have also been proposed and can guide the selection of exposures [20, 21]. Once the research question and set of exposure variables have been clarified, a considerable amount of pre-processing of the data is required.

### Handling missing data in the exposures

Missing data is problematic in an exposome context that examines exposures jointly because the number of complete cases may decrease as the number of included exposures increases. The use of imputation techniques is therefore recommended. Multiple imputation is commonly used to handle missing data in epidemiological studies, and Rubin’s rule can be used to combine coefficient estimates obtained from each imputed dataset [22]. Applying multiple imputation to large datasets involves additional difficulties that were previously described [23]. It is recommended that imputation models include no more than 15–25 predictors, since adding more predictors usually provides little gain and can lead to problems of convergence due to predictors collinearity [24].

### Dealing with exposure values below the limits of detection and quantification

For exposures that are measured through biochemical assays, some values may be below the limit of detection (LOD). The LOD is the lowest quantity of an exposure that can be detected by a specific method. A commonly used approach consists in replacing all values below the LOD by a fixed value such as the LOD, half the LOD or LOD/√2 [25]. While single substitution could be acceptable when the proportion of values below the LOD is low (e.g., < 5%), this method introduces bias in the results as the proportion of values below the LOD increases [26]. It is therefore recommended that values below the LOD are imputed using imputation approach for left-censored missing data (e.g., using the *imputeLOD* function available in the rexposome R package) [27, 28]. However, despite the good performance of the imputation methods, exposures with a high proportion of values below the LOD (e.g., > 80%) should be either not used or dichotomized into detected/undetected. Exposures measured through biochemical assays may also have values below the limit of quantification, which is the lowest quantity of an exposure that can be detected with a stated accuracy and precision. Exposures with a high proportion of values below the limit of quantification should be carefully interpreted.

### Correcting for measurement error of the exposures

An important issue in an exposome study concerns exposure misclassification, especially when the degree of measurement error differs from one exposure or exposure family to another. Assuming two exposures *A* and *B* are associated with an outcome and that exposure *A* has larger misclassification than exposure *B*, an exposome study ignoring exposure misclassification will more likely observe an association with the outcome for exposure *B* than for *A*, hence inducing differential power to detect associations. Ideally, the quantification of the measurement error for each exposure, typically through their intraclass correlation coefficient, would be available and a exposure measurement error correction method could be applied. However, this cannot be computed if no repeated biospecimen are collected, at least in a subset of the study population and the literature cannot always provide relevant intraclass correlation coefficient estimates since these need to be assessed from comparable studies, typically in terms of study population and of biospecimen collection time points. Intraclass correlation coefficient for some non-persistent chemicals have been recently reported [27]. Previous studies have described methods that jointly correct for measurement error and perform variable selection, but they are mostly complex to implement, not available in standard statistical software and not applicable to all types of regression models or to all settings [29–31]. A simulation study showed that two measurement error models (simulation extrapolation and regression calibration) limit attenuation bias due to exposure misclassification. A posteriori disattenuation can also be applied, dividing the effect estimate by the intraclass correlation coefficient of the corresponding exposure [32].

## Describing the exposome

Besides the summary statistics of each exposure, the exposome can be described in terms of its correlation structure, dimensionality, and variability. Gaining insight into this will allow improved analysis and interpretation of the associations with determinants and health outcomes.

### Correlation structure of the exposome

The correlation structure of a large set of exposures has been described using data from the Spanish INMA birth cohort and from the US NHANES and LIFE studies [17, 33–35]. More recently, the HELIX project has also described the correlation structure of the exposome, using over 200 environmental exposures that were assessed in pregnant women and later in their children in 6 European birth cohorts [19]. In these previous studies, the correlation coefficients were stronger within than between families of exposures. This may suggest that findings from epidemiological studies focusing on a single family of exposures may be less confounded by unmeasured exposures from other families, although this can vary on a case-by-case basis. Strong correlations between specific pairs of exposures from the same family were reported, although, particularly in the HELIX project, most correlations were low or moderate (the median correlation value observed within families was only 0.2). The exposome correlation structure seems also to vary across spatially and temporally distributed populations [19]. Thus, the exposures correlation structure needs to be evaluated in each exposome study, since it is largely influenced by the setting and by the set of exposures and exposure families included.

The correlation structure of the exposome may have implications in the exposome-health associations due to the difficulty to untangle the exposures truly affecting the health outcome from their correlated exposures, thus increasing the probability of obtaining false positives. A simulation study conducted by the HELIX and EXPOsOMICS projects showed that, due to the correlation within the exposome, the linear regression-based statistical methods that were investigated were only moderately efficient to differentiate true predictors from correlated covariates [36]. Therefore, to ensure a correct use of statistical methods and appropriate interpretation of the results, it is crucial to first explore the correlations between the exposures under study. For sets of exposures that exhibit absolute correlations higher than 0.9 and therefore are assumed to provide the same information, we recommend to select only one exposure since it would be very difficult to separate their effects unless very large sample sizes are available. Heat maps and circos plots have been developed to visually display the correlations [17, 34, 37]. Another tool to visualize the complex relations between exposures is a network analysis, in which exposures that are close together in the network are more correlated than more distant ones [19]. We should note that the correlation structure might be influenced by how the exposures were assessed or constructed and by the presence of measurement error. Exposures obtained using the same methodology, in the same biological matrix or constructed from the same variables may show greater correlation compared to those obtained by distinct approaches.

### Dimensionality of the exposome

Data-driven dimension reduction techniques, such as principal component analysis or factor analysis, can be used to describe patterns of exposure within the exposome. These techniques allow capturing the variance of many exposures in a smaller set of independent components or factors, each of them being composed of exposures that tend to occur simultaneously in the population. The results may vary according to the technique used, the number of components/factors retained and the loading value chosen as threshold [38]. Clustering approaches, such as partitioning, hierarchical, or model-based clustering, can be used to identify mutually exclusive groups of subjects sharing a similar pattern of exposure. Such clustering models assume that the study population is made of distinct groups of individuals sharing similar characteristics in the observed variables [39].

Multicenter studies pose special challenges for identifying exposome patterns, because usually the center will be the main driver of such patterns (e.g. in the same center, subjects will have similar diet or similar air pollution). Thus, cluster analyses may end up identifying centers as the clusters. One may attempt to remove center effects before performing cluster analysis, but that might eliminate existing exposure variation between centers [18].

These techniques can be applied either using all exposures or within each exposure family. The latter strategy might allow a better interpretability of the resulting patterns/clusters, but will not take into account and highlight the between-family variability. Obtaining exposome patterns/clusters of subjects instead of using all single exposures might facilitate the analyses and interpretation of the associations with determinants and health outcomes and reduce the problem of multiple testing. However, in exposome analyses including several exposures, it might be difficult to summarize the data in a reasonable number of patterns/clusters. In the HELIX project, due to the large number of exposures and low correlations observed between them, the exposome revealed to be high dimensional and difficult to summarize in a few principal components. Ten principal components explained 45% and 39% of the total variance in the pregnancy and childhood exposome, respectively, while 65 and 90 components were required to explain 95% of the exposome variability [19]. In studies using specific subsets of exposures, namely urban or chemical exposures, a reasonable number of principal components was obtained, reinforcing that summarizing the data might be easier for a smaller subset of exposures and within certain exposure families [15, 18].

Dealing with multiple imputation in dimension reduction or clustering techniques might be complicated. In principal component analysis or factor analysis, different components/factors with different interpretations can be obtained in each imputed dataset, which can be complex to combine. One suggested approach to deal with missing data in dimension reduction techniques consists in estimating the covariance matrix on the complete data (i.e. in each pair of exposures’ complete data), and then perform principal component analysis or factor analysis on this matrix [40]. Bayesian model-based clustering techniques can automatically handle missing data without having to use imputations. Bayesian techniques may not be feasible with high-dimensional or large sample settings due to high computational costs. In such cases, an alternative is a framework that integrates multiple imputation in cluster analysis, which has been previously described [41].

### Variability of the exposome

Unlike the genome, the exposome changes over time, which complicates its characterization. The levels of exposure might vary over time due, for instance, to changes in individual behavior (e.g., change in diet), in the outdoor environment (e.g., change of address), and in the governmental policy (e.g., restriction in use of bisphenol A in some countries). Large within-subject temporal variability is a common issue for non-persistent chemical contaminants as they can have short half-life in the human body. As a result, for many non-persistent chemicals, a few dozen samples seem to be required to accurately assess exposure over periods encompassing several trimesters or months [19, 27, 42]. A recent study, characterizing the personal external exposome, including air pollution, traffic-related noise, natural outdoor environments, ultraviolet radiation and levels of physical activity, of pregnant women and children in eight European cities, suggested that the assessment of these personal exposures requires monitoring from one day to more than one year, depending on exposure due to high variability between and within cities and participants [43].

The between-subject variability of the data observed for a given exposure may be influenced by the method of data collection. Differences in the variability were observed in the INMA pregnancy exposome dataset between the exposures measured through biomarkers and those derived from geospatial models [17]. Biomarker measurements showed higher between subject variability, which may be due to incorporating information regarding both prevalence in the environment and personal behavior.

## Assessing the determinants of the exposome

The exposome is specific of each individual but many parts of the exposome are shared between subgroups of the population due to shared determinants such as diet, physical activity, mobility, social and ethnic factors. Environmental inequality, which is the differential exposure to environmental factors between groups within a population, may have important health implications. The environmental inequality might differ by geographical setting. The HELIX project has previously reported that the urban exposome among pregnant women seems to be socially determined, with considerable differences among European cities [18]. Pregnant women of low socio-economic position were exposed to higher levels of environmental hazards in some cities, but not all, which may contribute to inequalities in child health and development. Other studies, embedded in the HELIX project but also using NHANES, INMA and Korean data, also showed that exposure to environmental contaminants seems to be determined by socioeconomic indicators, with both lower and higher socioeconomic groups incurring into high exposure levels [15, 44–46]. Assessing the determinants of the exposome will improve the understanding on the populations at higher risk of exposure to certain hazards and ultimately at higher risk of adverse health outcomes.

## Assessing exposome-health associations

The association between exposures and health has traditionally been assessed in studies focused on a single or a limited number of exposures. Besides providing only a fragmented view of exposome-health associations, results from these approaches may suffer from confounding due to unmeasured exposures, selective reporting and publication bias. This may be overcome by using a more holistic and systematic exposome approach. Insight into the relationship between the exposome and health outcomes can be obtained by relying on several methods, which have been previously described [47, 48]. A classification of the methods has been proposed and categorizes them into one of three different groups: variable selection, dimension reduction, and grouping of observations [48]. Analyzing several exposures together in an exposome context also allows identifying synergies or combined effects of groups of exposures that might confer an overall risk that differs from that derived from each exposure separately. Specific frequentist and Bayesian methods to analyze combined effects of exposures related to health risk have been previously reviewed [47].

We give here a non-exhaustive description of three groups of methods used to assess exposome-health associations: (1) single exposure approaches such as environment-wide association study (ExWAS), (2) variable selection techniques such as deletion-substitution-addition (DSA) algorithm, elastic net (ENET) or graphical unit evolutionary stochastic search (GUESS) algorithm, and (3) dimension reduction techniques such as sparse partial least squares (sPLS) regression. Unsupervised analyses for grouping of observations, such as cluster analyses, have already been briefly described in a previous section of this paper about the dimensionality of the exposome and thus will not be further discussed here. Also, details about the supervised techniques for grouping of observations can be found elsewhere [48].

### Single exposure approaches

One of the first studies using an exposome approach conducted an ExWAS to study type 2 diabetes mellitus using data from the NHANES [49]. In an ExWAS, a large number of exposures are successively and independently tested for their association with the outcome (only adjusting for potential confounders), using an analogous statistical approach to that of genome-wide association studies. Thus, there is no control for between-exposure confounding. Although not ideal, for practicality, and due to the large number of exposures involved in exposome studies, the same set of confounders is usually used for all exposures. A correction for multiple comparisons is further applied to limit false positive results. Various methods exist and can be divided into Family-wise error rate (FWER) and False discovery rate (FDR) methods. Among the most popular methods used in epidemiologic studies, we can cite the Bonferroni procedure and the Holm procedure (both FWER methods), or the Benjamini and Hochberg procedure (FDR method). However, one limitation of these methods is that they assume independence of tests and can be highly conservative when this assumption is violated [50]. In an exposome context, assuming this assumption is questionable since exposures may not be independent, e.g., in the case of a common source or chemical metabolites resulting from a single parent compound, or in the case of highly correlated exposures in the outdoor environment [18]. To counter this limitation, additional procedures exist, among which, one similar to the Bonferroni procedure (which consists in dividing the significance level α by the number of tests M; α_corrected_ = α/M), has been proposed: the idea is to change the value of M according to the “effective number of tests” determined from the correlation structure of the data [50, 51]. While several methods have been proposed for estimating the effective number of tests in genomic research, their performance in an exposome context has not yet been addressed [51]. Additionally, in case of multiple outcomes, multiple testing correction should take the multiplicity of both exposures and outcomes into account. Thus far, to the best of our knowledge, no suitable multiple testing correction method has been assessed that simultaneously handle the multiplicity of exposures and outcomes in an exposome context. Exposures that are significantly associated with the outcome after correcting for multiple testing may be subsequently included in a multiple regression model. This two-step approach is referred to as ExWAS-multiple regression. Unlike ExWAS, it allows to correct exposure-health associations for potential co-exposure confounding. Volcano and Manhattan plots may be used to display the associations obtained from the ExWAS approach. This approach is computationally efficient, can handle various types of outcome data, interactions and adjustment for covariates and is readily available in statistical software, namely in the rexposome package in the R software. In a simulation study mimicking a realistic correlation structure of exposure variables in the INMA birth cohort, the ExWAS underperformed the other methods in terms of false discovery proportion but displayed the largest sensitivity [36]. When ExWAS was followed by a multiple regression step, the problem of false discoveries was improved, but the sensitivity was low, indicating that only a small proportion of true predictors were captured. ExWAS analyses can easily integrate multiple imputed datasets. Most software/software tools are able to automatically conduct a regression analysis in each of the imputed datasets and combine the results while incorporating uncertainty due to imputations.

#### Variable selection techniques

These techniques are predictive methods that seek for a subset of exposures that are related to the outcome. Initially, all exposures are candidates, so they have the potential to control for between-exposure confounding. Due to its predictive nature, these methods may not be accurate for etiologic analysis, since an exposure potentially related to the outcome may not be selected if another highly correlated exposure is already selected and thus does not confer any additional improvement to the prediction. Examples of variable selection techniques include the DSA algorithm, ENET and GUESS algorithm.

The DSA algorithm was used, for instance, in recent studies within the HELIX project looking at the associations between the early-life exposome and childhood lung function and between the urban pregnancy exposome and birth weight [13, 16]. The DSA algorithm is an iterative model search algorithm that relies on deletion (removing one variable from the model), substitution (replacing one variable from the model by one that was not yet included in the model) or addition (adding one new variable to the model) moves to find the optimal set of variables that minimizes the root mean square error. The final model is selected based on cross-validation [52]. Due to the reliance on cross-validation, repeating the variable selection process could lead to different sets of exposures retained and therefore we recommend to run a minimum of 50 DSA models. Then, a potential procedure to have a final model would be as follows: (1) run a multi-exposure regression model including exposures selected in at least 5% of the DSA models (a different % cut-off might be applied); (2) exclude non-significant exposures one-by-one following the order of the frequency of selection until the final model that includes only significant exposures. We can argue that a non-significant exposure might improve the prediction of the outcome or confound another association and thus there is less consensus regarding the latter step. In simulation studies, DSA showed high sensitivity and low false discovery rate, and showed good performance in the ability to capture interaction terms [36, 53]. Pre-selection of candidate exposures (i.e. keeping only one exposure among highly correlated ones) may be useful to prevent problems with the DSA algorithm. In the HELIX project, it was defined that only one exposure would be included as candidate among exposures with correlations > 0.8. The DSA algorithm supports Gaussian, binomial, and multinomial outcome distributions (i.e., censored and counted data are not supported). It allows incorporation of nonlinear terms of predictors, interaction and adjustment for confounders. However, it is not possible to restrict interactions to a specific factor (e.g., sex) or exposure, and all pairwise interactions, but also quadratic terms, will be considered by the DSA. These limitations together with lack of statistical power has precluded the consideration of non-linear relationships or interactions in some previous work using the DSA algorithm [13, 53]. In practice, the DSA can be run using R after downloading the DSA package available on GitHub. All variables, which are not forced into the model, are candidates for selection. DSA models can suffer from a long computing time, although this is less problematic when using parallel computing. It is also not straightforward to combine this approach, or any other variable selection approach, with multiple imputation since a different set of variables may be retained in each imputed dataset. To overcome this, the analyses can be performed on an extended dataset obtained by stacking all imputed datasets, using weights to restrain to the initial sample size [54].

The ENET was previously used, for instance, to study the associations between multiple environmental contaminants and birth weight among three cohorts from Greenland, Poland and Ukraine and between several persistent organic pollutants in breast milk samples and infant behavioral problems [55, 56]. The ENET is a penalized regression model that combines advantages of both LASSO and Ridge regression. Briefly, the LASSO regression promotes sparsity and performs variable selection by shrinking the lowest regression coefficients, which correspond to the least informative predictors, to zero. The predictors with non-null shrunk coefficients are those found to be jointly associated with the outcome. The Ridge regression accommodates correlated variables and ensures numerical stability [57]. Generalized version of ENET accommodates linear, logistic, multinomial, Poisson and Cox regression models [58, 59]. In a simulation setting, this approach showed high sensitivity and a moderate false discovery rate [36].

The GUESS algorithm is a Bayesian variable selection technique that uses a search algorithm that is based on multiple chain genetic algorithms. It was proposed to explore complex genetic-association models and maximize genetic variant detection [60]. Similarly to the DSA algorithm, in a simulation study, GUESS showed high sensitivity and low false discovery rate [36]. However, accounting for confounders is not straightforward and can only be achieved by first fitting the outcome on the confounders, and then, fitting a GUESS model on the residuals.

### Dimension reduction techniques

Dimension reduction techniques, such as principal component analysis, have already been described in a previous section of this paper about the dimensionality of the exposome and also in a previous review [48]. We focus here, as an example of these techniques, on PLS approaches. The EXPOsOMICS project described the application of PLS approaches by investigating the effect of exposure to disinfection by products on inflammation [61]. The sPLS regression was previously used in studies aiming, for instance, to identify, from multiple contaminant exposures, exposure profiles associated with biomarkers of male reproductive function [62]. This approach builds latent variables (linear combinations of the predictors) in a supervised manner, i.e., using the outcome, and then regresses the outcome on the latent variables. The sPLS components do not only capture as much variance of the predictors as possible, but also focus on the variance that is relevant to the outcome of interest. It imposes sparsity when constructing the latent variables, so that they depend only on a subset of the original set of predictors, improving their interpretability [63]. This approach is computationally efficient, multicollinearity and over-fitting are no longer concerns but results may lack interpretability and the ability to adjust for confounders is limited. In a simulation setting, this approach showed high sensitivity and a moderate false discovery rate [36].

## Incorporating omics into exposome research

The internal exposome, defined as the internal endogenous processes including epigenetic, gene expression, inflammation, and metabolism, can be assessed through high-throughput molecular omics methodologies such as genome-wide DNA methylation, transcriptomics, proteomics and metabolomics [3]. Analytical platforms such as untargeted high-resolution metabolomics in blood specimens are extending the coverage of internal exposures of potential health significance, regardless of their exogenous or endogenous origin [64, 65]. In general, omics signatures may reflect both physiological responses to external exposures and internal signatures of health outcomes [66]. Omics data are heterogeneous in terms of their dimension, nature, complexity and stability/volatility. The strength and complexity of correlation structure are also heterogeneous across different types of omics data, varying from distance-driven correlation in the genome to more complex patterns in other omics, especially in metabolomics [66]. Omics data are also highly sensitive to matrix selection (e.g., blood, urine) and experimental conditions (e.g., time of sample collection, sample storage and analytical batches), and can therefore be affected by measurement error. For this reason, extensive data quality control prior to the main analysis should be undergone to remove potential technical noise hindering identification of biological effects. Techniques such as surrogate variable analysis aim to remove major variability in the dataset that is not related to the outcome of interest or known technical noise [67].

Omics can be considered (1) as the outcome and the external exposures as predictors, (2) as the exposures, fitting in the definition of the exposome as non-genetic causes of disease, or (3) as external exposome-health mediators and thus both external and internal domains should be analysed side by side, with emphasis on interaction between and within domains. To illustrate this last point, a study assessed the impact of the gut microbiome diversity on childhood asthma together with persistent organic pollutant exposure and found that gut microbial diversity did not mediate the observed association between environmental chemicals and asthma [68].

**Fig. 1:**
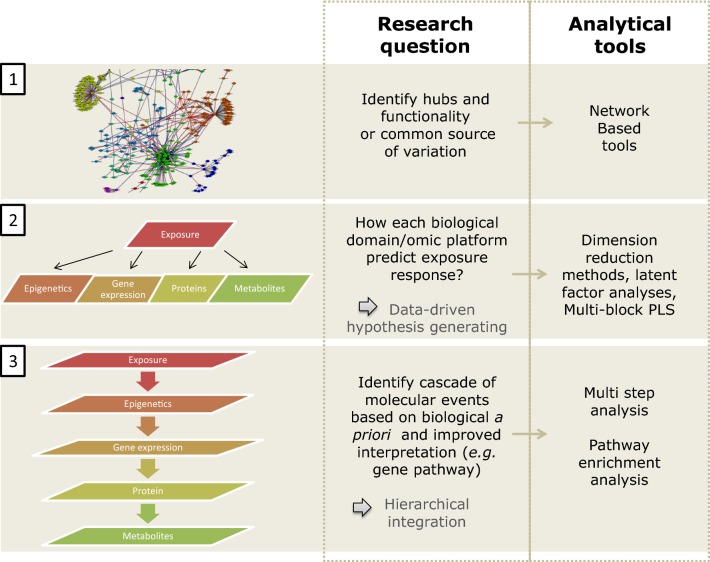
Specific omics tools that can be used in exposome research

Ongoing birth cohorts have increasingly access to large omics datasets characterizing the molecular phenotype in early life, before the development of clinical symptoms. Omics profiles are of interest as a phenotype outcome in itself without necessarily including clinical outcomes, and might be used as predictors of later disease risks. The analysis of omics profiles in relation to external exposome factors and/or clinical outcomes require the use of specific statistical tools due to the nature of the omics profiles (high dimensional, complex correlation structure and biological interpretation). We give here a non-exhaustive list of specific tools developed for omics data that can be adapted to study omics in the context of exposome research depending on the research questions (Fig. 1) [47, 61, 69–72]. Network based approaches in systems biology and medicine, including transcription factor binding, protein–protein interactions, metabolic interactions, genetic interaction and disease-disease association (diseasome) networks, have helped to interpret the behavior of molecules or diseases that are related, and to provide insights into their mechanisms. In an exposome context, network science enables organization of high dimensional omics data for visualization and information summary purpose such as to identify hubs of correlated exposures and to interpret systemic biological changes that associate with multiple exposures and health effects [73–76]. Networks can be used to study the relationships between exposures based on their correlation in a population or based on their chemical or toxicological properties, and therefore reveal grouping of exposures [77, 78]. This structure may be interpreted a posteriori through previous knowledge on the exposure sources (e.g., occupational exposure, consumer goods, diet), or in a biological context using KEGG pathways or mummichog for pathway enrichment analysis or adverse outcome pathway (AOPs). Inferred exposome networks based on the researcher collected exposure data can inform on new pathway to exposure in a specific cohort, for a specific age range or population subgroup that was not previously described [79]. If the network is based on collected omics data, the emerging hierarchical structure may not have been described yet in a general human population and may serve for grouping exposures into mixture and study combined effects on health. There are several limitations to these approaches, including the difficulty to link different types of exposures (e.g. chemical and outdoor urban exposomes) or different types of omics layers (e.g. methylation data with gene-level annotations versus metabolome data with microbiome/dietary related sources) to public libraries which are in general specific to one type of data. Supervised network alternatives, i.e. differential networks, can also be used to characterize differences in omics correlation patterns in subpopulations. This method consists of inferring first the individual network (i.e., exposed vs. non-exposed), define the metrics for the change in correlation between the two populations and finally identify significant changes in correlations. The less stable nodes may be the ones the most influential on their association to the outcome [80]. To explore the association between multiple external exposome constituents and omics profiles, taking into account the correlation structure of the omics, statistical inference can be done through dimension reduction techniques (PLS approaches) and variable selection techniques (with penalization or Bayesian Variable Selection) or a combination of both (sPLS). These techniques can also be used for more appropriate grouping of external exposures based on shared biological/physiological effects or shared source of exposure. A recent study applied a O2PLS approach in pregnant women from the Spanish INMA birth cohort to assess the association between multiple chemical exposures (> 30) and urine ^1^H nuclear magnetic resonance metabolomics (> 60). This allowed the identification of common route of exposures such as fish intake and oxidative stress of chemical exposures with known detrimental health effects [81].

When multiple omics profiles are available in the same individuals, cross-omics analyses investigate how exposure and/or outcome-related signals found at one molecular level correlate to those found at another level, gaining insight into the molecular cascades related to that specific exposure and/or outcome [82]. A UK twins study suggested that metabolomics combined with epigenetics could identify key molecular mechanisms in early development that lead to long-term physiological changes influencing human health and ageing [83]. There are several approaches for cross-omics analyses, that can be summarized briefly as (1) based on apriori biological knowledge by linking the omics layers through a common gene/pathway identifier and conduct pathway enrichment analyses or through looking for a candidate omics markers after explorative analysis in another omics layer or (2) without apriori biological knowledge through, for example, multi-block PLS model or canonical regression analyses [84].

Recently, dimension reduction techniques, such as sPLS, have also been extended to take into account functional grouping within the omics such as gene pathways, or metabolic function. For example, this tool was applied to identify the predictive power of prediagnostic levels of inflammatory markers in patients with B-cell lymphomas to select the most relevant protein groups in relation to disease status [85]. When considering the internal exposome as a mediator of the external exposome-health outcome, approaches such as the meet-in-the-middle approach may be useful, i.e., identifying biomarkers linking exposures and disease outcomes [86].

In general, longitudinal cohort studies have been applying omics technologies in nested case–control studies to assess specific health outcomes or to study one particular external exposure effect such as tobacco smoking or arsenic. To our knowledge, there is no previous study that presents a more comprehensive exposome approach by taking into account multiple external exposures and multiple omics signatures.

## Sample size in an exposome context

Statistical power in an exposome context is limited by the multiplicity of exposures tested and the correction for multiple testing performed. This is even a bigger concern if low to moderate association sizes are expected for the exposures, and when a substantial proportion of concentrations below LOD are present. These issues can be partly overcome by increasing the sample size of the study. A previous study estimated the required sample size to perform an ExWAS approach of endocrine disrupting chemical biomarkers in relation to male fertility outcomes [14]. Since the effect sizes are typically low and the biologically significant sizes for these exposures is not known, the authors assumed a null effect size distribution and took the 95th percentile effect size as a threshold of important effect size. Under these conditions, an ExWAS approach combined with FDR multiple testing correction would require a sample size of 1000–2000 subjects to deal with 100 exposures and achieve power of 80% to detect the 95th effect sizes. This study also showed that, in comparison with Bonferroni correction, the FDR correction allowed to rely on a smaller sample size, although much larger than the required to provide sufficient power when a single exposure is considered.

## Challenges for future exposome statistical analyses

Ideally, statistical methods used in exposome research should be capable of handling a large set of time-varying exposures from different domains, while considering correlation structures and accounting for multiple comparisons. To date, it remains difficult to efficiently untangle the exposures truly affecting the health outcome from correlated exposures and to identify synergistic effects between exposures. The incorporation of the multilevel structure assuming the three interlinked general and individual external and internal domains, and of the causal structure between exposures within and across domains into an exposome analysis is another complexity still to be addressed. Pregnancy and birth cohort studies have collected a large amount of longitudinal data on exposures. However, there are no clear guidelines on how to conduct longitudinal analyses with exposome data. The longitudinal aspect further increases the dimensionality of the data and forces the analyst to take more decisions. For example, one can model changes, trajectories of both exposome and outcome, cumulative exposure, lagged effects, or look for windows of susceptibility, among others. Due to the temporal structure, potential time-varying sets of confounding factors should be taken into consideration. Future simulation studies should assess the performance of several statistical exposome methods in the context of longitudinal modelling. The EU Child Cohort Network, which is established by the LifeCycle Project, represents an unique opportunity to address these statistical issues and provides an important framework to conduct replication studies in exposome research.

## Conclusions

The exposome is a promising field of research that will help identifying exposures that impact health and disease across the lifespan. Despite considerable methodological advances, estimating the association between a large set of exposures and health is still challenging. On-going and future projects on the exposome, including theoretical and methodological studies, will be crucial to explore ways of overcoming the limitations of the current methods and ultimately allow a better understanding of the human exposome.
